# Travel times and distances to health services in Australia

**DOI:** 10.1038/s41597-025-06381-y

**Published:** 2025-12-18

**Authors:** Michaela Filipcikova, Louisa Jorm, Sebastiano Barbieri

**Affiliations:** 1 The Hopkins Centre, Griffith University, Brisbane, QLD Australia; 2 Centre for Big Data Research in Health, University of New South Wales, Sydney, NSW Australia; 3 Queensland Digital Health Centre, The University of Queensland, Brisbane, QLD Australia

**Keywords:** Health services, Public health

## Abstract

Geographic accessibility, the ability to physically reach healthcare services, is a critical determinant of healthcare equity and outcomes. In Australia, over 42,000 individuals residing in inner regional, outer regional, remote, or very remote areas lack access to any primary healthcare service within a 60-minute drive. Such limited access is associated with reduced service utilisation and poorer health outcomes. This study examined national patterns of healthcare accessibility by estimating average driving times and distances between geographic areas (Mesh Blocks, Statistical Areas Level 2, Postal Areas and Modified Monash Model areas within each Australian State and Territory) and selected healthcare services. Using the Open Source Routing Machine, we calculated travel times to the nearest public and private hospitals, emergency departments, general practitioners, bulk-billing general practitioners, and pharmacies. The resulting dataset enables consistent healthcare accessibility comparisons across service types and geographic regions in Australia. The Python code used in this analysis is publicly available and can be adapted to generate similar datasets for other countries.

## Background & Summary

Geographic accessibility is a key aspect of healthcare delivery^1,2^. It refers to the ease with which individuals can physically reach healthcare services and is typically assessed by examining factors such as population distribution (demand), the availability of healthcare facilities (supply), and transportation options^3^. A research report by the Australian Royal Flying Doctor Service from September 2020^1^ determined that more than 42,000 Australians, or almost 10% of people living in inner regional, outer regional, remote or very remote Australia, have no access to any primary healthcare services within a 60-minute drive time. When looking at individual primary healthcare providers, 65 000 people have no access to a GP within a 60-minute drive. Issues associated with longer travel time to healthcare services include lower usage of these services^4–6^, lower survival rates^5–7^, and even increased risk of death by suicide^8^.

Mesh blocks (MBs) are the smallest geographic units defined by the Australian Bureau of Statistics (ABS) and serve as the foundation for the larger regions within the Australian Statistical Geography Standard (ASGS). The ABS currently defines 368,286 MBs covering the whole of Australia without gaps or overlaps^9^. Counts of the total usual resident population are available for each MB based on the 2021 Australian Census of Population and Housing^10^. Statistical Areas Level 1 (SA1s) are geographic units composed of whole MBs, and SA1s can be aggregated to form Statistical Areas Level 2 (SA2s). Most SA2s have a population range of 3,000 to 25,000 people and are designed to represent communities that interact socially and economically. According to the most recent ABS release from July 2021, there are 2,473 SA2s covering all of Australia, with no gaps or overlaps, including 19 non-spatial special purpose codes (e.g., ‘Outside Australia’ or ‘No Usual Address’)^11^.

SA2s are typically the smallest geographic units used for the release of non-Census ABS statistics, including Health and Vital Statistics data. In this study we also considered alternative geographic units which may be available in Health data, namely Postal Areas and Modified Monash Model (MMM) areas. Postal Areas are MB-based approximations of general definition postcodes by the ABS, where MBs are allocated to Postal Areas based on the largest population contribution^12^. The MMM is a classification of area remoteness and population size ranging from MM 1 (major city) to MM 7 (very remote)^13^.

This study aimed to estimate average driving times and distances to closest healthcare services for people living withing specific geographic areas (MBs, SA2s, Postal Areas, MMM areas within each State and Territory) across Australia. Travel duration estimates are used to better reflect real-life travel effort, rather than relying on straight-line distances. This study builds upon our previous work^14^ which estimated travel times between each SA2 and each hospital in Australia, as well as the shortest travel time to any hospital, averaged across residents of a SA2. This work has since been adapted to other contexts^15–17^. In this study we used similar methods to our previous work but included different types of geographic areas and healthcare services. We differentiate between public and private hospitals, since the latter are accessible only to individuals with private health insurance or those who choose to pay out of pocket. The provided services also differ, with private hospitals specialising in elective procedures and typically not operating emergency departments. Besides hospitals, this study includes emergency departments, general practices (GPs), bulk-billing GPs, and pharmacies. Bulk-billing GPs provide healthcare at no cost to eligible individuals by accepting the Medicare benefit as full payment and billing Medicare (i.e., the publicly funded universal health care insurance scheme in Australia) directly, so patients have no out-of-pocket expenses. This model makes access to GP services affordable to the general population. Pharmacies were also included, as distance to them can affect people’s ability to maintain a continuous supply of medications. Further, the recent expansion of pharmacies’ prescription rights in some Australian states allowing pharmacists to prescribe the contraceptive pill and treatments for common conditions may encourage more individuals to rely on pharmacies rather than GPs^18^.

With the release of this dataset and related software code into the public domain we aim to facilitate research on access to healthcare services, their utilization and outcomes. Its availability will encourage consistency across studies investigating the impact of geography on health disparities in Australia (including between Indigenous and non-Indigenous groups) and the methods are applicable to computing travel durations and distances to health services in other countries.

## Methods

Health service locations were provided by Healthdirect Australia, a national health advice service, upon request for the purpose of this project^19^. The dataset provides a complete list of health facilities across Australia, including 643 public hospitals, 258 private hospitals, 579 emergency departments, 7,120 GPs, 1,282 bulk-billing GPs, and 6,353 pharmacies across Australia. Verification of the geographic coordinates of 10 randomly sampled health services in each Australian State and Territory did not indicate any inaccuracies. K-d trees – binary search trees for organising k-dimensional data^20^ - were used to store health service coordinates and allow efficient retrieval of the 10 providers closest to each MB centroid, in terms of straight-line distances.

MBs for Australian States and Territories were downloaded from the Australian Bureau of Statistics (ABS) website in shapefile format^21^. The centroids of each MB were computed using the QGIS geographic information system software^22^. Besides centroid coordinates, the resulting table contained the SA1, SA2, and State or Territory of each MB. This table was then linked with population data^21^, Postal Area data^12^, and MMM data^23^.

OpenStreetMap data for Australia were obtained from the Geofabrik download server^24^. These data are updated daily and made available free of charge. The cartographic data were used to initialise a local instance of the Open Source Routing Machine (OSRM) HTTP server. Driving times and distances from each MB centroid to 10 nearby public and private hospitals, GPs, bulk-billing GPs, emergency departments, and pharmacies were computed using OSRM’s Table service^25^. Travel duration, distance and coordinates of the service with the shortest driving time to each MB were saved in an output table.

**Fig. 1:**
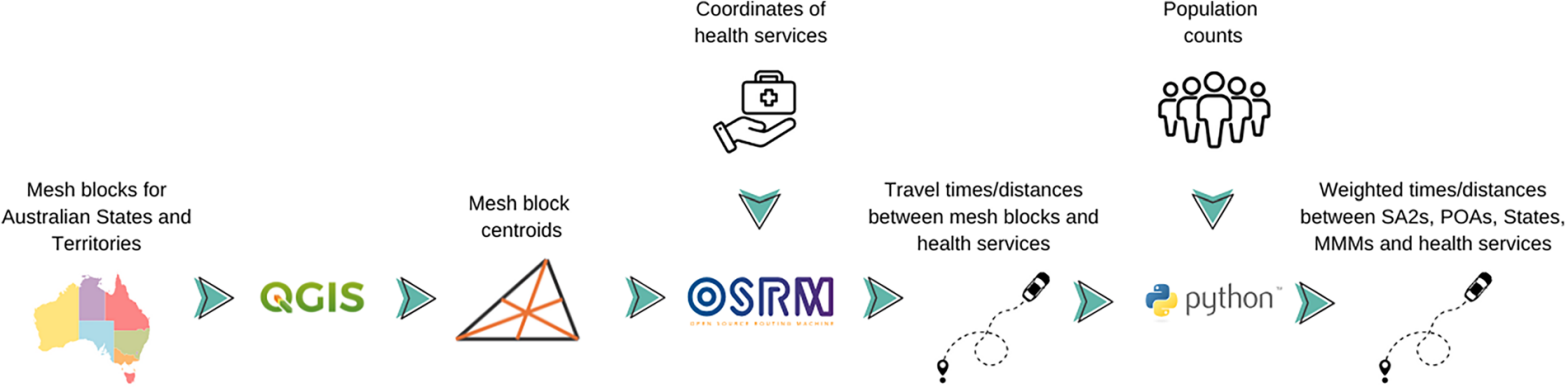
Schematic overview of the process used to compute travel times and distances between geographical areas and health services. SA2: Statistical Area Level 2; POA: postal area.

Finally, population-weighted average driving times and distances to the closest health service were computed for SA2s, Postal Areas, individual MMM areas within Postal Areas, MMM areas within each State and Territory. Alternative geographic groupings can be easily determined starting from the MB-based table. These computations were carried out using Python version 3.10.4. An overview of our approach is presented in Fig. 1.

## Data Records

Specific details regarding the size and format of the data records are provided in Table 1, and the records themselves are available on figshare^26^. The *mb_2021_centroids* table comprises data on the 2021 MB codes (MB_CODE21) and their geographic coordinates (longitude, latitude). The *mb_2021_distances* table contains the 2021 MB codes (MB_CODE21) along with corresponding population count (Person), SA2 regions (SA2_CODE21), State or Territory code and name (STE_CODE21, STE_NAME21), Postal Area (POA_CODE_2021), Modified Monash Model classification (MMM2019), travel times and distances to each healthcare service (hospital_public_duration, hospital_public_distance, hospital_private_duration, hospital_private_distance, gp_duration, gp_distance, gp_bulk_billing_duration, gp_bulk_billing_distance, emergency_duration, emergency_distance, pharmacy_duration, pharmacy_distance) and coordinates of the nearest health service (*_longitude, *_latitude). The remaining tables *weighted_averages_sa2, weighted_averages_poa, weighted_averages_poa_mmm, weighted_averages_ste_mmm* contain population-weighted averages for each health service grouped by SA2s, Postal Areas (_poa), States and Territories (_ste), Modified Monash Model categories (_mmm). Lastly, ‘australia-latest’ contains the OpenStreetMap data that were used for computing travel times and distances to health services^24^.

**Table 1:** Format and size of data records.

Name	Format	Size [KB]	Num. Rows
*mb_2021_centroids*	csv	95,566	368,286
*mb_2021_distances*	csv	84,944	368,256
*weighted_averages_poa*	csv	634	2643
*weighted_averages_poa_mmm*	csv	816	3422
*weighted_averages_sa2*	csv	599	2463
*weighted_averages_ste_mmm*	csv	14	58
*australia-latest*	osm.pbf	841,079	NA

csv: comma-separated values; pbf: OpenStreetMap protocolbuffer binary format; NA: not applicable.

## Technical Validation

We showed in previous work that the OSRM server provides routing results comparable to commercial services such as Google Maps, with duration differences generally under 5 minutes^13^. OSRM server does take into account possible ferry services but may underestimate travel times if no route is found (e.g., there are currently no passenger ferries to King Island in Tasmania or Lord Howe Island in New South Wales [NSW]).

**Fig. 2:**
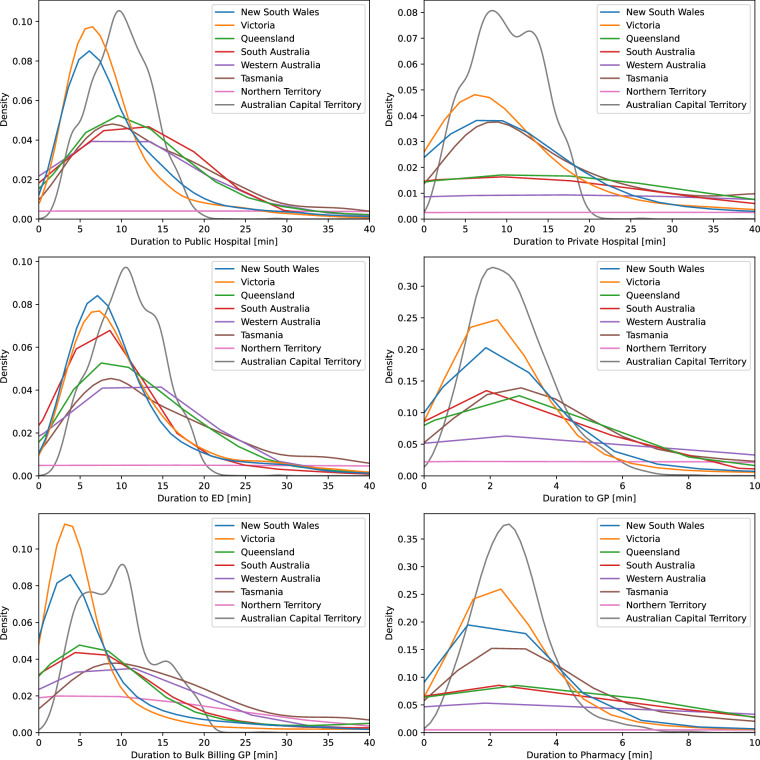
Distribution of shortest travel times to selected health services in Australian States and Territories.

**Fig. 3:**
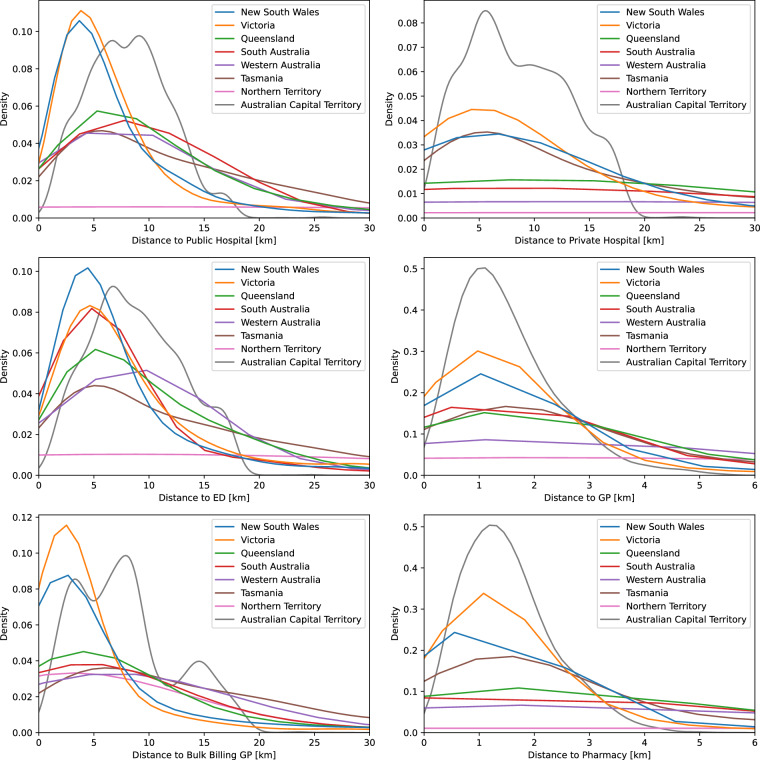
Distribution of shortest travel distances to selected health services in Australian States and Territories.

**Fig. 4:**
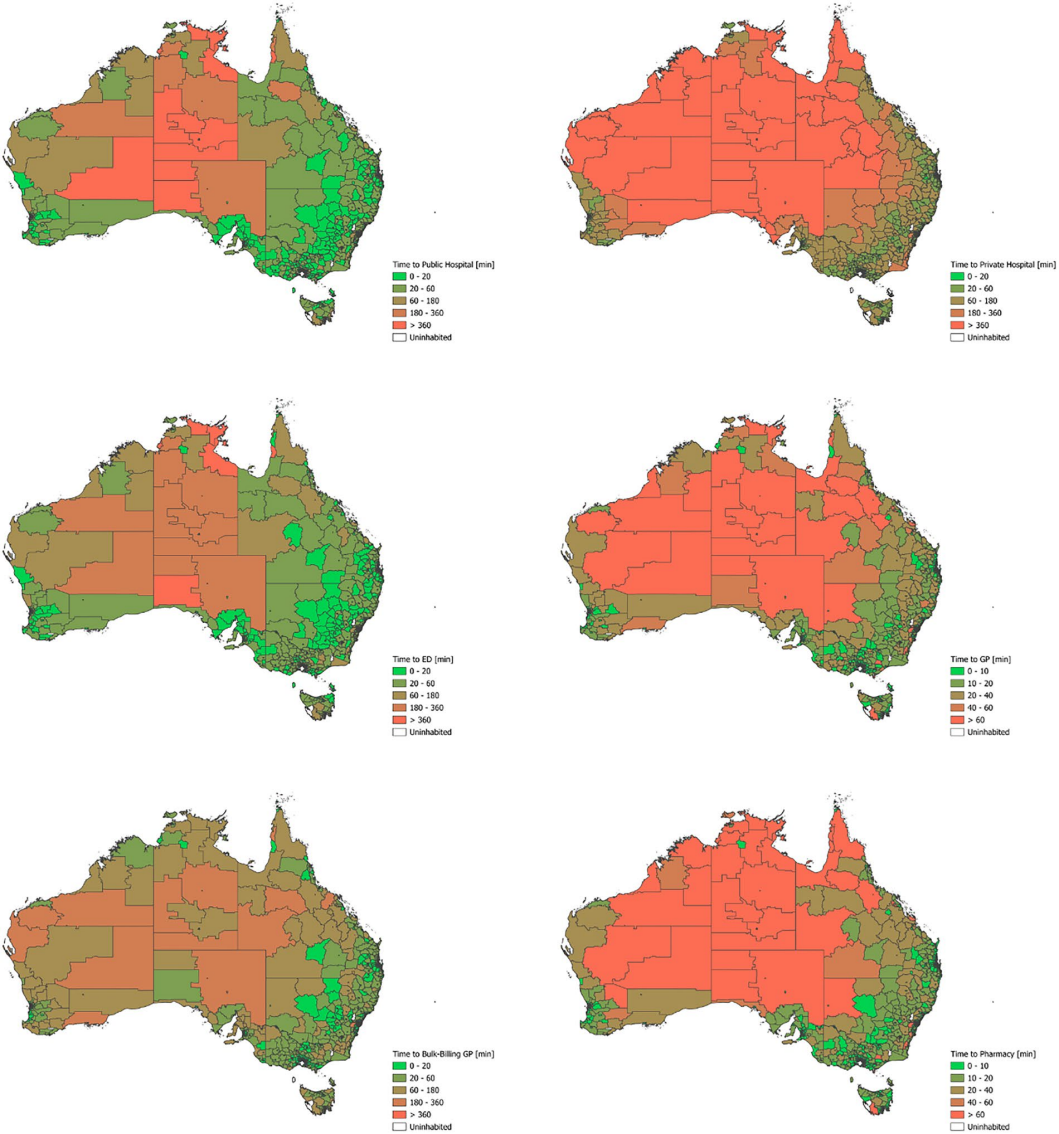
Map of Australia divided into Statistical Areas Level 2 (SA2s), color-coded based on the shortest travel time to each type of health service.

Figures 2 and 3 show kernel density plots depicting the distributions of shortest travel times and distances to the considered health services based on population-weighted MBs, grouped by Australian State/Territory and service type. Figure 4 displays maps of Australia divided into SA2s, with each area color-coded based on the shortest travel time to each type of health service. The greater density of GPs and pharmacies results in most residents being able to access these services within a ten-minute drive. The plots and maps make use of different scales to reflect differences in accessibility between health services. A notable feature in the density plots is the shorter tail of travel time and distance distributions in the Australian Capital Territory (ACT), reflecting its small geographical size. The relatively long travel times to a public hospital/ED in the ACT are likely due to the low density of public hospitals in the ACT (only two). Further, the tri-modal distribution of travel times to bulk-billing GPs in the ACT may reflect their clustering in three main areas: North, South-West, and South-East of the city centre. The relatively flat kernel density distribution of shortest travel times in the Northern Territory reflects both the geographic sparsity of health facilities and the wide variability in travel times across the region.

## Data Availability

The data related to this study is available at 10.6084/m9.figshare.30018415.

## Code availability

The Python code related to this study can be accessed at https://github.com/MFilipcikova/hs-distances-aus.
